# Canine models of inherited retinal diseases: from neglect to well-recognized translational value

**DOI:** 10.1007/s00335-024-10091-y

**Published:** 2024-12-30

**Authors:** Valérie L. Dufour, Gustavo D. Aguirre

**Affiliations:** 1 Division of Experimental Retinal Therapies, Department of Clinical Sciences, School of Veterinary Medicine, University of Pennsylvania, Philadelphia, PA, USA

## Abstract

Large animal models of inherited retinal diseases, particularly dogs, have been extensively used over the past decades to study disease natural history and evaluate therapeutic interventions. Our group of investigators at the University of Pennsylvania, School of Veterinary Medicine, has played a pivotal role in characterizing several of these animal models, documenting the natural history of their diseases, developing gene therapies, and conducting proof-of-concept studies. Additionally, we have assessed the potential toxicity of these therapies for human clinical trials, contributing to the regulatory approval of voretigene neparvovec-rzyl (Luxturna^®^) by the US Food and Drug Administration (FDA) and the European Medicines Agency (EMA) for the treatment of patients with confirmed biallelic mutation-associated retinal dystrophy. In this review, we aim to summarize the clinical features of a subset of these diseases and reflect on the challenges encountered in integrating canine models into the translational pipeline.

## Introduction

### Background

In a series of seminal studies in the first half of the 1950’s, H.B. Parry demonstrated the value of the canine model for comparative studies of inherited retinal diseases (selected references include (Parry 1953, 1954). The veterinary ophthalmology specialty was just developing, and there was an increased interest in identifying and characterizing the many forms of inherited retinal diseases, particularly degenerations, that were being recognized clinically in dogs. Degenerations fell under the rubric Progressive Retinal Atrophy-PRA-and many anecdotal reports suggested that over 100 different dog breeds were affected with PRA. Because of advances in cell biology and physiology in the 1970’s to 1980’s, our lab became a leader in identifying different retinal disease models and establishing the structural, functional and, where possible, biochemical basis of the diseases. We expanded on the Parry studies in the Red Irish Setter (Parry 1953) by showing that the disease results from abnormal photoreceptor development, now termed rod-cone dysplasia 1 (*rcd1*) (Aguirre and Rubin 1975; Buyukmihci et al. 1980), and is caused by abnormal cGMP metabolism in the visual cells (Aguirre et al. 1978, 1982b). Other diseases soon were identified that affected the retinal pigment epithelium (RPE) (Aguirre and Laties 1976), rods (Aguirre et al. 1978), cones (Aguirre and Rubin 1975), or both rod and cone photoreceptors (Acland and Aguirre 1987; Aguirre et al. 1982a; Parkes et al. 1982).

The initial successes in identifying and developing these models for retinal disease studies prompted the National Retinitis Pigmentosa Foundation (RP Foundation; now Foundation Fighting Blindness) to provide partial pilot support to ensure that these models also would be made available to other vision scientists. Success with the pilot program made the RP Foundation lobby members of Congress to encourage the National Eye Institute/National Institutes of Health (NEI/NIH) to enhance this support through a contract mechanism open to all applicants. We were successful in the application process and received a 3-year contract to fund the research colonies and promote their use by vision scientists in the US and abroad. After renewal of another 3-year contract, the funding mechanism was changed in 1984 to a Cooperative Agreement mechanism with a 5-year funding cycle. The wide use of the resources and need to expand and house the research dogs in a centralized facility resulted in construction of a canine care and research facility at the University of Pennsylvania. The facility was finished in 1989 and funded jointly by the RP Foundation and the NEI.

### Dark clouds in the horizon

At the time the Cooperative Agreement was submitted for competitive renewal in 1989, the decision was made by the NEI to cut the funding cycle from 5 to 3 years, further reducing the 3-year funding by 40%, and designating the project for future review through the RO1 mechanism^1*^. The decision was justified partly because dogs were ‘too expensive’ to maintain, and there were no genomic studies ongoing that would enhance their use as an animal model for human retinal disease. In 1990, the opsin gene had been identified as a cause of autosomal dominant RP (Dryja et al. 1990). The reasoning used at the time was that with the development of knockout and other transgenic technologies, it would be relatively ‘easy and cheaper’ to create mouse models on demand for any known or suspected retinal disease gene mutation. Thus, canine models would be superfluous for research.

### Growth of tools and genomic resources for canine retinal disease studies

The move of the canine retinal disease studies from cell biology/biochemistry/pathology to molecular studies was essential if progress was to be made. As the lines of dogs were quite inbred and lacked polymorphisms for linkage studies, Dr. Gregory Acland, a senior research investigator within our laboratory started a program of outcrossing affected dogs for 3 autosomal recessive diseases to genetically normal dogs of various other breeds; the progeny, all obligate heterozygotes, would be backcrossed to the affected dogs and the progeny scored for disease (homozygous affected) or no disease (heterozygous non-affected). In several prior studies we had developed functional and structural methods (Acland and Aguirre 1987; Aguirre et al. 1982a; Buyukmihci et al. 1980) to definitively score the phenotypic outcomes.

Unbeknown to us, at the same time we were developing pedigrees for mapping inherited traits of interest, Drs. Elaine Ostrander and Jasper Rine had been developing a set of polymorphic markers for linkage mapping of diseases of interest to their laboratory. However, they lacked extensive and informative pedigrees to do this work. On the other hand, we had pedigrees but no markers. A collaboration was born!!!

A project that started ~ 1995 soon proved successful. Within less than 2 years, the first linkage map of the dog genome was completed (Mellersh et al. 1997), and in parallel, an effort was directed to physically separate the canine genome by making canine-rodent hybrid cell lines (Langston et al. 1997). From then on progress was rapid and 1 year later the first autosomal disease in dogs, progressive rod-cone degeneration (*prcd*), was mapped to CFA9 (Acland et al. 1998). Progress continued apace and in a matter of 2 years the whole-genome radiation hybrid map was reported (Priat et al. 1998), a canine BAC library was made (Li et al. 1999), and the linkage and radiation hybrid maps integrated (Mellersh et al. 2000). It was only a short matter of time before the dog genome was fully sequenced (Lindblad-Toh et al. 2005) after an earlier effort for lower coverage sequencing (Kirkness et al. 2003). The tools that developed from these advances allowed for high quality genomic studies in the species. It is fair to say that the impetus for this work was directly or indirectly attributable to Dr. Elaine Ostrander (now at the National Human Genome Research Institute of the National Institutes of Health) who was outspoken but persuasive in her support for the dog as a model organism for phenotype, behavior and other studies (Chase et al. 2005, 2009; Ostrander 2012; Ostrander and Kruglyak 2000; Parker et al. 2004, 2010; Shearin and Ostrander 2010).

Despite the progress made, the late 1990’s-early 2000’s sometimes were difficult because many organizations and some journals still considered the dog as an orphan species unworthy of study. NEI provided targeted funding for studies to develop retinal cDNA microarrays for human, non-human primates, rodents and others, but specifically excluded dogs. Likewise, the NIH-sponsored CIDER program at Johns Hopkins’ Center for Inherited Disease Research did not include the dog among the species supported for genotyping. Multiple letters to NEI/NIH to address this shortcoming went unanswered. Even the Marshfield genotyping center initially excluded dogs from their services. This policy was reversed in mid 2000, but the number of genotyping that they required then as a minimum was 10,000, which was too large for the small canine pedigrees. That number was subsequently reduced, and we took advantage of this program with great success (Goldstein et al. 2010; Kukekova et al. 2006).

NIH study sections often were not receptive to the advantages of the canine model for translational research. A June 2000 summary statement to a NEI R01 proposal, indicated that ‘*The advantage of the dog over the mouse is clearly its larger size*, *which allows surgical procedures and analyses that may not be possible with the limited tissues in the mouse. However*, *most of the studies proposed … could probably be done just as easily and more cheaply and rapidly in the mouse*’. In addition, other critiques included ‘*The dog model is original but*, *although it might be of great help for surgical methods*, *it is mostly a drawback for the experiments presented here’*. Lastly, *‘Although this application represents a very thorough and scientifically sound approach to gene therapy*, *it appears that the use of the dog model will*, *at this point*, *very seriously hamper the success of the experiments*’. Soon after, we showed that the review was premature and incorrect (Acland et al. 2001).

And finally, some journals were also not receptive to our submissions for canine studies. Our March 2001 submission to *Mammalian Genome* (Integrated Meiotic, Radiation Hybrid and Comparative Mapping of the XLPRA Interval of the Canine X Chromosome) among others was rejected, and the general feeling within our group was that the only mammalian species worthy of the journal was the mouse. Fortunately, that policy, too, was changed, and *Mammalian Genome* now has been publishing high impact canine studies for many years.

### Grand slam home run

The canine models came into their own with our 2001 Nature Medicine publication showing that gene therapy restored vision in the canine model of childhood blindness (Acland et al. 2001). This was the first clear demonstration that AAV-mediated gene therapy of the retina had a positive outcome on a disease. The longer term stability of this treatment was confirmed in a subsequent study (Acland et al. 2005), and three competing groups at Children’s Hospital of Philadelphia, Scheie Eye Institute-Philadelphia/University of Florida, and Moorfields Eye Hospital in London, raced towards clinical trials. The product has now been commercialized and is used in many countries on a worldwide basis. Other successful gene therapy efforts came out of our laboratory, for example for achromatopsia (Komaromy AM, 2010) and X-linked retinitis pigmentosa (Beltran et al. 2012, 2015) among others. As well, many other groups have made major strides in developing the canine model as a therapeutic test bed for inherited retinal degenerations (Annear et al. 2011, 2013; Bainbridge et al. 2015; Beckwith-Cohen et al. 2023; Mowat et al. 2013; Occelli et al. 2023; Petersen-Jones et al. 2017, 2019).

The following sections and Table (Table 1) briefly summarize these disease models for which a corrective gene therapy has been validated, and IND-enabling^2*^ studies completed, are currently ongoing, or soon to be initiated, within our research facility. When applicable, pitfalls with human pre-clinical trials will be discussed.

## Disease of the retinal pigment epithelium (RPE)

### RPE65 mutation - Leber Congenital Amaurosis (LCA)

An abstract published in 1983, described a litter of Briard puppies affected with pendular nystagmus, and functional night blindness (Riis et Aguirre, 1983). A few years later, similar findings were reported in Sweden where a litter of Briard dogs was identified with congenital stationary night blindness, closely resembling a form of congenital stationary night blindness in human (Narfström et al. 1989). The disease was characterized by absence or severely reduced retinal function for both types of photoreceptors, rods and cones, as measured by the electroretinogram (ERG), without funduscopic changes at early ages (Narfström et al. 1989). Some dogs also exhibited nystagmus. Histopathological studies revealed the accumulation of vacuolated cytoplasmic inclusions in the retinal pigment epithelium (RPE) (Aguirre et al. 1998; Nilsson et al. 1992; Wrigstad et al. 1992). The condition was later found to result from a 4-bp mutation at the putative exon 5 of *RPE65* gene (Aguirre et al. 1998; Veske et al. 1999). This mutation led to inactivation of the RPE65 enzyme, an isomerohydrolase, that plays a crucial role in the RPE retinoid circle (Jin et al. 2005).

A little over two decades ago, restoration of visual function and vision was achieved following subretinal injection of an adeno-associated vector carrying full length RPE65 cDNA (Acland et al. 2001). Since then, numerous studies in RPE65-mutant dogs have reported recovery of function in the treated eye (Acland et al. 2001, 2005), even when intervention was performed at an advanced age and disease stage (Annear et al. 2013). But for how long? Unfortunately, in human trials, the results were less impressive and not sustained long-term when intervention occurred at a later stage of disease. A decline in function and retinal structure was observed in RPE65 patients despite therapeutic intervention (Jacobson et al. 2015). This prompted our group to investigate the impact of intervention at even later stage of disease, with a longer post-intervention observation period, to better mimic what might occur in humans. The study revealed that approximately 60% of the retinal photoreceptors need to be present at the time of intervention to achieve significant and sustained preservation of the retinal structure (Gardiner et al. 2020). Thus early treatment, before there is extensive loss of photoreceptors and their nuclei, favor better long-term outcomes.

### Bestrophinopathies

A canine bestrophinopathy, initially described as “*canine multifocal retinopathy*” (cmr), was first reported in the Great Pyrenees breed (cmr1) by Grahn (Grahn et al. 1998). Subsequent studies by our group at the University of Pennsylvania identified two additional mutations within the same gene (*BEST1*) that result in a similar phenotype. These mutations were found in the Swedish Lapponian Herder breed (cmr3) (Zangerl et al. 2010) and in the French Coton de Tuléar (cmr2) (Guziewicz et al. 2007; Grahn et al. 2008). Collectively, these conditions are referred to as ‘canine bestrophinopathies’. The *BEST1* gene is expressed at the basolateral aspect of the RPE cells where it’s function is posited to be a Ca^2+^- activated chloride channel (Hartzell et al. 2008). It plays a crucial role in the development of apical microvilli, RPE cell homeostasis, and lipid metabolism (Guziewicz et al. 2017).

Regardless of the mutation, the disease phenotype in homozygous or compound heterozygous dogs, is characterized by a retina-wide micro-detachment (Guziewicz et al. 2018) and the formation of multifocal retinal detachments, of varying sizes, often filled with a red/brown tinged fluid (Guziewicz et al. 2007, 2011, 2016, 2017). In advanced stages, known as the vitelliruptive and atrophic stages, the lesions diminish in size and result in outer nuclear thinning and scarring, and loss of retinal structure, as observed via invivo imaging (data not published). The retina-wide micro-detachment was found to be modulated by light (Guziewicz et al. 2018; Wu et al. 2024), and this phenomenon was subsequently used as an outcome measure to assess successful restoration of RPE-photoreceptor interface homeostasis following corrective gene therapy (Guziewicz et al. 2018). A comprehensive review of the future for BEST1 gene therapy is available (Amato et al. 2023).

## Disease of the cone photoreceptors

### Achromatopsia

In the late 1960s and early 1970s, a naturally occurring disease leading to day blindness was identified in the Alaskan malamute breed (Aguirre et Rubin, 1974). The later identified mutation affects the cyclic nucleotide-gated (CNG) channel subunit beta 3 (CNGB3) (Sidjanin et al. 2002). Affected dogs are born with day blindness, displaying rod electroretinogram (ERG) function comparable to that of wild-type dogs, but no detectable cone function. Notably, no funduscopic changes are observed in these dogs, even up to at least four years of age. More recently, our group has identified a similar phenotype in the German shepherd and Labrador retriever breeds (Tanaka et al. 2015) which have different mutations in the alpha subunit of the channel *CNGA3*).

Another large animal model for achromatopsia was identified by a group in Israel, in sheep. Both models (Banin et al. 2015; Gootwine et al. 2017; Komaromy et al., 2010, 2013; Ye et al. 2017) were used for proof-of-concept studies for gene augmentation therapies, with results up to six years following treatment (Ofri et al. 2018).

When transitioned to human clinical trials, the impressive results achieved in our large animal models were not fully replicated. While the studies showed that the vectors were safe and well tolerated, there was a significant lack of recovery of photopic visual function. A three-year phase I clinical trial using AAV8-hCAR-CNGA3 showed consistent improvement in best-corrected visual acuity (BCVA) and contrast sensitivity in the treated eye (Reichel et al. 2022). However, due to slight improvement in BCVA in the untreated contralateral eye and the small number of treated patients, statistical significance could not be reached when comparing the treated and untreated eyes.

Similarly, a lack of significant efficacy was observed in patients treated with AAV8-hCAR-p.hCNGB3 up to 24 weeks post-treatment (Michaelides et al. 2023). Notably, some patients did exhibit improvement in color vision, reduced photophobia, and better outcomes in vision-related quality of life questionnaires. These findings underscore the need for continued development of additional sensitive and quantitative endpoints.

Compared to the results in large animal models, these human outcomes may highlight the necessity of targeting areas outside the traditionally focused fovea and macula where cones may be less affected by the disease process. Additionally, the promoter used in human clinical trials (hCAR) was not the most effective in dog models (Komáromy et al. 2010). Lastly, while older *CNGA3* sheep responded to therapy in a comparable way than younger animals (Banin et al. 2015), older *CNGB3* mutant dogs required pre-treatment intervention with CNTF-mediated photoreceptor deconstruction to exhibit a positive therapeutic response (Komáromy et al. 2013). Unfortunately, this approach has been ineffective when applied to human patients (Zein et al. 2014).

Part of the reason for the less-than-spectacular results in human clinical trials compared to those in sheep, dog and mouse models (Sarfare et al. 2015; Zhang et al. 2020) is that therapeutic intervention in animals were preceded by extensive studies examining the cell biology, physiology, and biochemistry of the diseases. This in-depth understanding helped inform the therapeutic strategies in animal models. However, such comprehensive knowledge may not have been available for some of the human clinical trials. Going forward, advanced technologies such as adaptive optics and optoretinography, among others, will provide valuable insights to guide future interventions for this group of diseases (Jiang et al. 2024).

## Disease of the rod photoreceptors

### Rhodopsin autosomal dominant retinitis pigmentosa (RHO-adRP)

One of our previous collaborators, Dr. Gregory Acland, discovered that some English mastiffs undergoing repeated eye examinations exhibited varying levels of retinal degeneration at different age. This observation led to the identification of a naturally occurring T4R dominant mutation within the rhodopsin gene of affected dogs (Kijas et al. 2002). The mutation disrupts the first consensus glycosylation site on Asn2 in the protein, but does not impede the trafficking of newly synthetized opsin to the outer segment. However, the mutation affects the stability of the protein when it lacks 11-cis retinaldehyde chromophore such as it occurs after bleaching the dark-adapted retina with light (Zhu et al. 2004). As a result, dogs with mutant rhodopsin are extremely sensitive to light. A similar susceptibility to light-induced damage has been observed in some rhodopsin mutations in humans (Cideciyan et al. 2005) and also is found in the T17M mouse model where the second consensus glycosylation site is eliminated (White et al. 2007).

This light sensitivity in the canine model has proven invaluable for therapeutic intervention studies as it allows for experimental control over the onset of rod photoreceptor loss and the modulation of degeneration severity by exposing the dogs to varying levels of light (Iwabe et al. 2016; Sudharsan et al. 2017). When protected from bright white light since birth, and kept under dim red light conditions, the retinas of RHO-mutant dogs remain normal and comparable to WT (Cideciyan et al. 2005; Dufour et al. 2017). Surgical intervention on the eyes requires operating under near infrared conditions, so as to avoid triggering a retinal degeneration (Komáromy et al. 2008).

The challenges at developing a gene therapy for RHO-adRP, a dominant disease that is caused by numerous distinct mutations within the same gene, was recently overcome by our group. Development of a single AAV vector that combines knockdown of the endogenous *RHO* mRNA and its replacement with a codon-modified resistant copy of human *RHO* cDNA provides a potential novel gene therapy approach for RHO-adRP that is mutation-independent. When tested in the T4R RHO dog model, subretinal delivery of this vector prevented light-induced photoreceptor cell death, and showed excellent retinal structure preservation and function within the treated area (Cideciyan et al., 2018b).

## Disease of the rod and cone photoreceptors

### RPGR-X-linked retinitis pigmentosa (RPGR-XLRP)

Three naturally occurring X-linked mutations have been reported in dogs (Zhang et al. 2002; Kropatsch et al. 2016); two of which were identified by our group. The first mutation named XLPRA1, is a 5 nucleotide deletion in the C-terminal domain of the *RPGR*-ORF15 that truncates the protein (Zhang et al. 2002). It was identified in the Siberian husky and Samoyed breeds. This mutation allows for normal development and function of both rods and cones up to approximately 1 year of age, after which slow degeneration begins. Clinically, it results in a juvenile-onset disease that starts peripherally and primarily impacts rod function, with robust preservation of the cones and central retina observed until later in life (Beltran et al. 2012, 2014).

A 2-nucleotide deletion in *RPGR*-ORF15 was found in the Miniature schnauzer/mongrel cross (Zhang et al. 2002; Beltran et al. 2006; Murgiano et al. 2019), and the condition named XLPRA2. In this model, rod development is altered very early, around 4 weeks of age, leading to abnormal maturation and function prior to degeneration (Beltran et al. 2006). Treatment with an *RPGR* gene augmentation approach at both early and late stages of the disease led to the prevention or arrest of disease progression, maintaining robust retinal structure and function of both rods and cones within the treated area (Beltran et al. 2012, 2015, 2017). The more rapid natural disease history and the shorter time frame required to assess therapeutic outcomes led us to favor the second type of X-linked PRA over XLPRA1.

Safety and efficacy studies were carried out by our group in the canine model (Dufour et al. 2020; Song et al. 2020). Despite natural disease history studies in patients suggesting that positive effects of therapeutic intervention would take ~ 4 years for the rods and ~ 6 years for the cones to be detected following gene therapy intervention (Cideciyan et al. 2018a), early phase I clinical trials results were encouraging. These trials demonstrated an excellent safety profile and improvement in visual fields beginning one month post-treatment, maintained over the 6-month study duration (Cehajic-Kapetanovic et al. 2020). Positive treatment effects persisted when the study period was extended to 12 months, with no dose-limiting toxicities reported and some patients showing improved and sustained retinal sensitivity and low-luminance visual acuity (Von Krusenstiern et al. 2023). However, the progress collapsed mid-2021 when Biogen released an online communication announcing that the phase II/III clinical trial had been aborted due to a lack of sustained efficacy. In contrast, studies in the canine model using a different vector have shown retention of structure and function for over 6 years following treatment (unpublished data). It is possible that a different vector than the one used in the Cehajic-Kapetanovic et al. clinical trial would prove more efficacious (Cehajic-Kapetanovic et al. 2020).

## NPHP5-Leber congenital amaurosis

The most recent mutation for which corrective gene therapy has been published in our lab was identified in the American pit bull terrier. The mutation involves a C insertion in exon 10 of *NPHP5* (*IQCB1*) resulting in a 12-amino-acid frameshift and truncates ~ 45% of the C-terminal amino acids (Goldstein et al. 2013). Clinically, some affected dogs exhibit nystagmus, and very early-onset day blindness. Functionally, photopic ERG responses are unrecordable, and scotopic ERG responses are markedly abnormal by 6 weeks of age and extinguished by 14 weeks of age. Structurally, a relatively well-preserved visual streak and area centralis can be observed, as illustrated by in vivo OCT imaging up to 33 weeks of age (Downs et al. 2016). Histological studies revealed that cone outer segments fail to develop, despite the population number of cones in the retina being comparable to that of WT. This explains the non-functionality of the cones, even though they are present in appropriate numbers (Downs et al. 2016).

Regardless of disease severity, gene therapy intervention has proven extremely successful in restoring retinal function and visual behavior, as well as reforming cone outer segments and stabilizing rod outer segments when treatment is administered at an early disease stage (6 weeks), with a follow-up of 6 months (Aguirre et al. 2021). Subsequently, the therapeutic window for intervention has been extended to include mid- to late-stage disease, demonstrating functional and visual recovery of both rods and cones up to 5 years post-treatment (Beltran et al. 2022). Notably, when treatment was performed at the most advanced disease stage, only cone-mediated function and vision were recovered. Furthermore, functional MRI (fMRI) studies confirmed the restoration of only cone-specific cortical function following gene therapy intervention, which was performed at mid-stage disease (34 weeks prior to imaging), when stimulating the treated and untreated eyes separately (Taskin et al. 2024). Taken together, these results are very encouraging for restoring cone function and vision in late-stage disease of NPHP5-LCA.

## Conclusions

Over the years, dogs have been proven to be an invaluable large-animal model for studying IRDs. Researchers have identified many naturally occurring mutations that closely model those found in human patients. This feature offers a significant advantage over rodent models, enabling the characterization of the natural disease history, the impact of the specific mutations on protein interactions, cellular architecture, or function, and the evaluation of therapeutic interventions. Additionally, the presence of a fovea-like region within an area of increased cone density along the visual streak, has been well characterized (Mowat et al. 2008; Beltran et al. 2014). This region is particularly useful for studying the topography of retinal diseases, their impact, and responses to treatment. Moreover, the globe size in dogs is similar to that of human, providing an excellent opportunity to perform surgical intervention, in-vivo imaging, and to test novel ocular-oriented surgical devices (Kwok et al. 2024). Important limitations of the dog model include the high costs associated with maintaining a dog colony, the slower development of the animals, and the sometimes slower progression of the disease, which may not allow for the rapid turnover of results achievable in mouse models.

Canine models have played a pivotal role in the success of corrective gene therapy for inherited retinal diseases in humans. Understanding the natural history of the disease in both animal models and human patients is crucial for setting realistic expectations and outcome measures for clinical trials. Data derived from non-clinical studies should be utilized to guide clinical trials, tailor appropriate outcome measures, and suggest precautionary measures that should be considered for each specific disease.

## Figures and Tables

**Table 1:** Abbreviated list of canine IRDs with validated corrective gene therapy studies carried out in our lab

Condition	Gene	Breed(s)	References
Non-clinical studies and clinical trials completed/market approval			
Leber Congenital Amaurosis-RPE65	*RPE65*	Briard	(Acland et al. 2001, 2005; Aguirre et al. 1998; Narfström et al. 1989, 1999)
Non-clinical studies toxicity/efficacy completed/Clinical trials ongoing			
X-Linked Retinitis Pigmentosa	*RPGR*	Miniature schnauzer/Mongrel cross Siberian husky	(Zhang et al. 2002; Beltran et al. 2006, 2012, 2015, 2017; Dufour et al. 2020; Song et al. 2020)
Achromatopsia	*CNGB3*	Alaskan malamute, German shorthair pointer	(Komáromy et al. 2010, 2013; Ye et al. 2017)
Non-clinical toxicity/efficacy studies in progress			
Best Disease	Best	English mastiff, Coton de Tuléar, Laponian herder	(Guziewicz et al. 2007, 2018)
Rhodopsin autosomal dominant retinitis pigmentosa	RHO-T4R	English mastiff	(Kijas et al. 2002; Cideciyan et al. 2005; Cideciyan et al., 2017, 2018b; Iwabe et al. 2016)
Non-clinical proof of concept studies			
Leber Congenital Amaurosis - NPHP5	NPHP5	American pit bull terrier	(Downs et al. 2016; Aguirre et al. 2021)
Congenital stationary night blindness	LRIT3	Beagle	(Kondo et al. 2015; Miyadera et al. 2022; Takahashi et al. 2023)

## Data Availability

No datasets were generated or analysed during the current study.

## Footnotes

1 * R01 grants are awarded to support a discrete, specified, circumscribed project to be performed by the named investigator(s) in an area representing his specific interest and competencies. The review process is highly rigorous and involves a comprehensive evaluation by a panel of experts.

2 * Investigational new drug (IND)-enabling studies are exploratory studies to evaluate the safety and efficacy of a drug. These trials include non-clinical in-vitro and in-vivo studies to assess pharmacodynamics, pharmacokinetics, and toxicology of the drug studied.
