# Correction: Canine models of inherited retinal diseases: from neglect to well-recognized translational value

**DOI:** 10.1007/s00335-025-10108-0

**Published:** 2025-02-11

**Authors:** Valérie L. Dufour, Gustavo D. Aguirre

**Affiliations:** Division of Experimental Retinal Therapies, Department of Clinical Sciences, School of Veterinary Medicine, University of Pennsylvania, Philadelphia, PA USA

**Correction: Mammalian Genome**

10.1007/s00335-024-10091-y

In this article the author Gustavo D. Aguirre should have been denoted as a co-corresponding author.

The original article has been corrected.

